# Strategies to Optimize Participation in Diabetes Prevention Programs following Gestational Diabetes: A Focus Group Study

**DOI:** 10.1371/journal.pone.0067878

**Published:** 2013-07-04

**Authors:** Kaberi Dasgupta, Deborah Da Costa, Sabrina Pillay, Mirella De Civita, Réjeanne Gougeon, Aaron Leong, Simon Bacon, Stephen Stotland, V. Tony Chetty, Natasha Garfield, Agnieszka Majdan, Sara Meltzer

**Affiliations:** 1 Department of Medicine, McGill University, Montreal, Quebec, Canada; 2 Department of Exercise Science, Concordia University, Montreal, Quebec, Canada; 3 Department of Psychology, McGill University, Montreal, Quebec, Canada; 4 Department of Pathology and Molecular Medicine, McMaster University, Hamilton, Ontario, Canada; Iran University of Medical Sciences, (Islamic Republic of Iran)

## Abstract

**Objective:**

We performed a qualitative study among women within 5 years of Gestational Diabetes (GDM) diagnosis. Our aim was to identify the key elements that would enhance participation in a type 2 diabetes (DM2) prevention program.

**Research Design and Methods:**

Potential participants received up to three invitation letters from their GDM physician. Four focus groups were held. Discussants were invited to comment on potential facilitators/barriers to participation and were probed on attitudes towards meal replacement and Internet/social media tools. Recurring themes were identified through qualitative content analysis of discussion transcripts.

**Results:**

Among the 1,201 contacted and 79 eligible/interested, 29 women attended a focus group discussion. More than half of discussants were overweight/obese, and less than half were physically active. For DM2 prevention, a strong need for social support to achieve changes in dietary and physical activity habits was expressed. In this regard, face-to-face interactions with peers and professionals were preferred, with adjunctive roles for Internet/social media. Further, direct participation of partners/spouses in a DM2 prevention program was viewed as important to enhance support for behavioural change at home. Discussants highlighted work and child-related responsibilities as potential barriers to participation, and emphasized the importance of childcare support to allow attendance. Meal replacements were viewed with little interest, with concerns that their use would provide a poor example of eating behaviour to children.

**Conclusions:**

Among women within 5 years of a GDM diagnosis who participated in a focus group discussion, participation in a DM2 prevention program would be enhanced by face-to-face interactions with professionals and peers, provision of childcare support, and inclusion of spouses/partners.

## Introduction

Gestational Diabetes (GDM) or ‘diabetes of pregnancy’ occurs in 2 to 7% of pregnant women [1], [2]. Generally resolving soon after delivery, it nonetheless signals a seven- fold increase in risk of maternal type 2 diabetes (DM2) within the subsequent 5 to 16 years [3], [4]. This partly results from lower physical activity levels and higher levels of excess weight: women with a GDM history are 40% more likely to be physically inactive and nearly twice as likely to be obese [5]. However, DM2 prevention is possible: in the American National Institutes of Health’s Diabetes Prevention Program (DPP), an intensive individualized intervention targeted at improving eating and physical activity habits led to a 50% reduction in diabetes incidence in the subgroup of women within 10 years of GDM [6].

Unfortunately, engaging women with a GDM history in DM2 prevention efforts is challenging [7]–[10] closer to the time of pregnancy. In one trial, women 6 weeks post partum were randomized [11] to test a comprehensive intervention (8 healthy-eating classes, 10 physical activity classes, 6 telephone-counselling sessions over 9 months) against usual care. However, participants attended fewer than four classes on average, and thus no benefits were demonstrated. In a second more promising trial, [12] a DPP-style intervention was delivered through four in-person and 13 telephone-based sessions. Compared to the control arm (i.e., written educational materials only), 16% more in the active trial arm attained the weight goal, although the between-arm difference was not conclusive.

There is a pressing need to determine the specific factors that could improve attendance and participation in diabetes prevention programs soon after a GDM pregnancy. This is because optimizing dietary and physical activity habits has the potential not only to prevent DM2 and cardiovascular disease in the long term, but also to prevent recurrent GDM, particularly if additional weight gain between pregnancies is minimized [13]. Therefore, we invited women within five years of a GDM diagnosis to a focus group discussion with the aim of delineating factors that could enhance participation and engagement in a DM2 prevention program.

## Research Design and Methods

### Recruitment Strategy

The study was approved by the McGill University Institutional Review Board, and participating institutions (McGill University Health Centre and Sir Mortimer Davis General Jewish General Hospital, Montreal, Canada). One previous qualitative study in women with GDM history [14] relied on advertisement-based recruitment. We instead instituted a *structured* recruitment strategy. Women previously followed at GDM clinics received up to three focus group participation invitation letters, signed by their physician (SM, NG, or AM). Those interested were scheduled at one of four possible discussion sessions that included one weekend morning, one weekday morning, and two weekday evenings.

### Focus Group Discussions

Written consent was obtained. Participants received $20 for transportation/parking-related costs and some refreshments. Two focus group discussions were bilingual (English and French), one was exclusively in English, and one exclusively in French. All were audio-taped and transcribed in their original languages.

An experienced moderator guided discussions (MDC; holds doctoral degree in psychology), with note taking by a co-moderator (SP; kinesiologist trained in qualitative methods). Both are women fluent in English and French. The interview guide [15] included (1) introductory remarks outlining the purpose of the discussion; (2) overview of informed consent and the purpose of audio-taping; (3) an explanation of moderator and co-moderator roles; (4) clarification of terms; (5) an outline of the ground rules; (6) an overview of a previous program as tested in adults with DM2, as discussed below; (7) four questions designed to elicit all the necessary information; and (8) debriefing. Two questions directly gauged participants’ impressions of the strategy as presented in terms of interest and barriers. Two others more generally queried potential facilitating factors, but included probes to gather perspectives regarding meal replacements and the usefulness of social media.

With respect to item (6), as a starting point for discussion, we described an intervention approach that we have pilot tested in adults with DM2 [16] that included meal preparation training (‘cooking lessons’) combined with nutrition education and pedometer-based self-monitoring. This approach has demonstrated clinically-important improvements in glycemic control (i.e., 0.3% reduction over 6 months in hemoglobin A1C) that correlate with small weight changes. Time-efficient, balanced meals were prepared in small groups under a chef’s supervision, with concurrent discussion with a dietitian. This was combined with pedometer-based self-monitoring [16]. Of note, neither the moderator nor co-moderator of the focus groups was involved in this latter study. The lead investigator (KD) described the intervention, and then left the discussion, so as not to influence its content.

### Focus Group Discussion Analysis

Qualitative content analysis of the focus group transcripts was performed by the moderator and co-moderator [15]. The transcripts were read, re-read, and text responses were independently coded according to which questions they addressed. Text segments were compared across the groups, seeking similar or repeated ideas. The two coders met to arrive at a consensus regarding their initial coding, and the coding manual was repeatedly modified with the reading of each transcript to accommodate for clarity and richness of ideas. The final step involved labeling identified themes for each question. Although the transcripts were coded in the original language (i.e., English or French), all quotations are presented in the English language herein, with French quotations translated to English.

### Participant Characteristics

Following discussions, participants were asked to complete a questionnaire by mail to ascertain demographic and psychosocial characteristics, and were asked to permit access to any glucose tolerance test results following pregnancy, as recorded in their electronic medical records.

## Results

**Figure 1:**
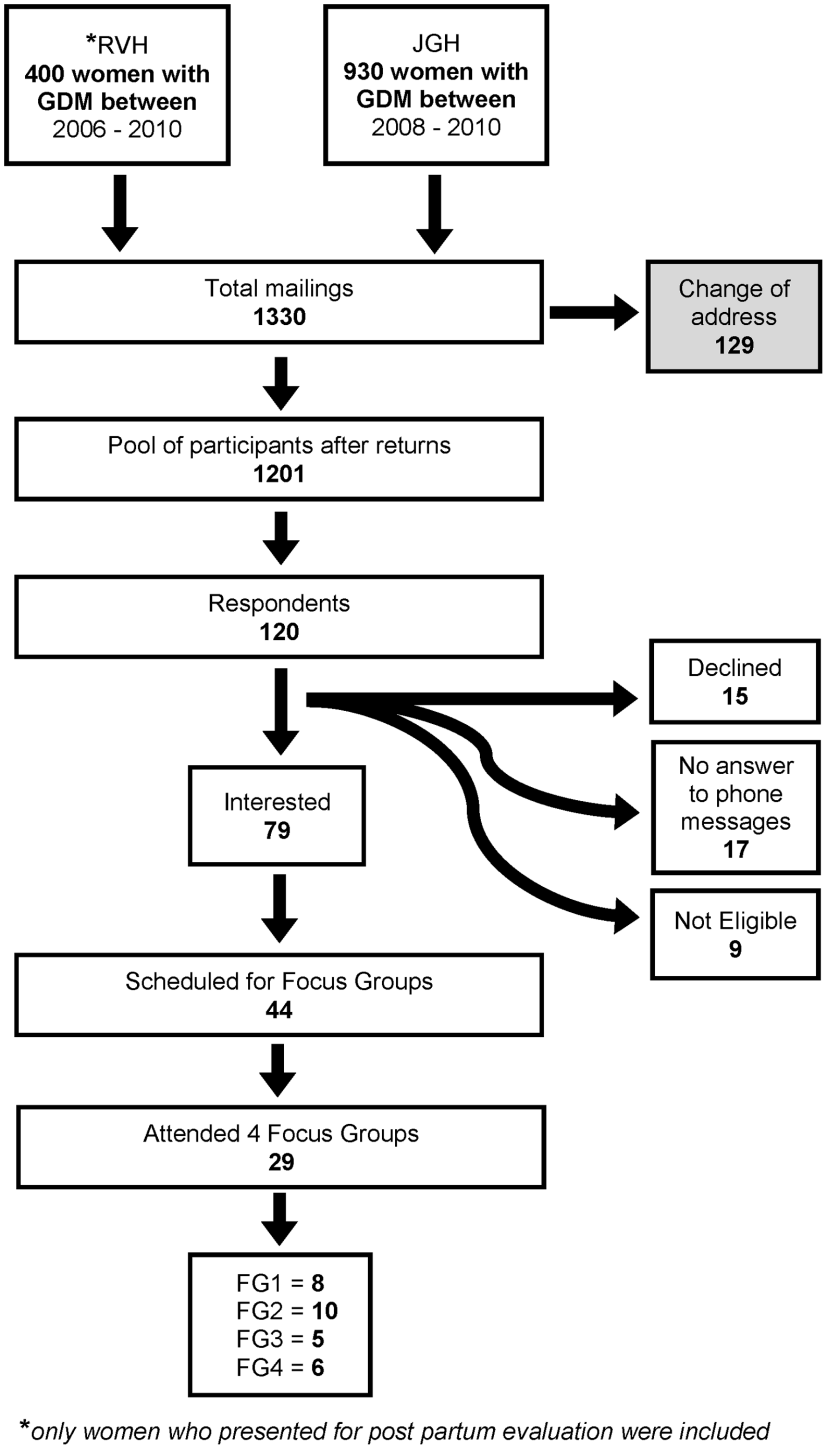
Participant flow.

Among 1,201 women who received invitations to participate (Figure 1), 120 contacted study personnel (10%). Among these women, 9 were not eligible (five did not have a GDM history, one had type 1 diabetes, one had diabetes following pancreatic surgery, one was pregnant, and one had developed DM2) and 15 were not interested (three cited distance from the study centre as a factor, two had a child sick at home, two reported they were too busy, and eight did not specify a reason). Seventeen had indicated interest on their original post card but subsequently did not respond to telephone messages. Among the 79 who were confirmed to be interested, 44 indicated that they would be able to attend a focus group on one of the dates proposed and were scheduled. Among these, 29 women actually attended one of the four focus group discussions (Saturday 14 April 2012 at 9∶30 AM; Wednesday 18 April 2012 at 8∶30 AM; Tuesday 21 June 2012 at 7∶15 PM; Thursday 26 June 2012 at 7∶10 PM). They were 40 years of age, on average (Table 1), all had completed education beyond high school, and over half were university-educated. Fifty-eight percent worked outside the home and 63% were of Europid origin. Regions of origin represented included Eastern Europe, Western Europe, South America, the Caribbean, and Asia. Roughly one third had a body mass index (BMI) above 25 kg/m^2^ prior to their first pregnancy, more than half had an elevated BMI at the time of the focus group discussion, and fewer than half were engaging in regular physical activity. Among the 13/29 who underwent 75 gram oral glucose tolerance testing within six weeks of delivery, all had biochemical evidence of elevated insulin resistance (i.e., 9 women with Matsuda index ≥3.0, 1 with HOMA-IR ≥3.6 and 11 with 1- hour PC ≥8.6 mmol/L) [17], [18].

**Table 1:** Focus group participant characteristics.

Age, mean (SD), years	40.3 (4.3)
Europid, N (%)	15 (63)
Employed, N (%)	14 (58)
University, N (%)	15 (63)
Pregnancies, Median (IQR)	2 (1, 3)
Number of pregnancies with Gestational Diabetes Mellitus, Median (IQR)	1 (1,2)
Prior Preeclampsia, N (%)	2 (8.3)
**Before first pregnancy**	
Prepregnancy BMI (SD), kg/m^2^	23.4 (5.0)
Overweight or obese before first pregnancy, N (%)	8 (33)
**Insulin resistance** **in last GDM pregnancy€**	
HOMA-IR mean (SD)	1.65 (1.22)
HOMA-IR ≥3.6, N (%)	1 (8)
Matsuda Index mean (SD)	5.87 (4.62)
Matsuda Index ≥3.0, N (%)	9 (69)
1 hr PC mean (SD), mmol/L	10.52 (1.88)
1 hr PC ≥8.61 mmol/L (%)	11 (85)
Elevated insulin resistance by ≥1 parameters, N (%)	13 (100)
**Current**	
BMI (SD), kg/m^2^	27 (6.5)
Overweight or obese, N (%)	13 (54)
Regular physical activity#, N (%)	10 (42)
Smoker, N (%)	3 (1.3)

24 of 29 participants completed an exit questionnaire and 13 had blood tests following pregnancy that permitted computation of a measure of insulin resistance.

# Regular physical activity defined as an activity performed at a moderate intensity for a total of 30 minutes throughout the day on most days of the week OR vigorous activity 3 times a week for 20 minutes at a time.

€ Homeostasis Model Assessment–Insulin resistance (HOMA-IR), Matsuda Index and 1 hour post cibum (1 hr PC) were calculated for 13 participants who underwent 75 g oral glucose tolerance testing with 4 time points at the McGill University Health Centre; HOMA-IR ≥3.6 imply possible hepatic insulin resistance if concomitantly overweight/obese; Matsuda Index ≥3.0 imply possible whole body insulin resistance (Stern SE et al., Diabetes 2005; http://mmatsuda.diabetes-smc.jp/english.html); 1 hr PC ≥8.61 mmol/L (155 mg/dl) is a marker of cardiovascular risk and insulin resistance (Bardini et al., Diabetes Care 2010).

The themes that achieved saturation across four focus groups are presented below, with key illustrative quotations. More quotations are provided in Appendix S1.

QUESTION 1: What would make someone like you take part in a program like this?

Seven themes emerged in the discussion that followed this question. Participants recognized the potential value of a meal preparation/nutrition education program (Theme 1: Potential Benefits from Nutrition/Culinary Education), expressing a desire to improve their knowledge of the optimal dietary intake after GDM, as exemplified by the following:

*Do you follow strictly (the plan) like you were pregnant or do you deviate from it a little because your body’s handling it differently… Am I going in the right direction?*

Underpinning the interest was a desire to prevent both DM2 and future GDM pregnancies, and to benefit from a behavioural change support system (Theme 2: Support for Lifestyle Change and Maintenance, with Prevention of Diabetes and Availability of Support System as Subthemes). Many were aware that GDM led to a higher risk of diabetes in the future:

*It is dormant, and that’s it. The older you get, the closer you get to forty – it comes out. You don’t realize it. But I realize that, my lifestyle has to change.*

Some emphasized the importance of sharing knowledge and experiences both with health professionals and with women like themselves:

*You need to get out, you need to go do stuff for yourself. I think part of the motivation that will get you kick-started is if you have people to talk to.*

Support at home from family members was deemed as critically important (Theme 3: Family Participation). Particularly crucial was buy-in and support from the partner/spouse to alter eating habits in the home. There was strong indication that the partner should be directly involved in any program:

*It’s nice that we’re being educated. I can tell the message to my husband. He can be supportive- but when I give this man brown rice, he looks at me funny. So I can explain to him really what’s going on but if he would hear it from elsewhere, maybe, it’ll be different.*

Moreover, participants recognized and emphasized the potential for a DM2 prevention program to have positive effects on the eating habits of all family members, particularly children.

*I think the kids too it’s important to learn from a young age, the proper eating habits. You might have something and it doesn’t always have to be sweet. Otherwise, later on, she’ll have diabetes. So I think it’s very important*

There was some general reflection on when the optimal timing of a DM2 prevention program should be (Theme 4: Right Timing/Opportunity for Intervention). Some suggested that the best time to initiate DM2 prevention efforts would be during pregnancy, while others believed that an intervention immediately following pregnancy would be best to maintain the improvements in eating habits they had achieved during pregnancy. A critical element that emerged was the need for child care support in order to attend DM2 prevention sessions (Theme 5: Availability of Child Care).

*You could offer services where you bring a child with you in a daycare or workshop, where you know that they are being taken care of.*

When probed on their views about how to integrate physical activity into their busy schedules, participants emphasized that any activity should be easily accessible (Theme 6: Awareness of Physical Activity Integration). Having access to a trainer as part of the program could help them get into the habit of incorporating physical activity into their daily lives.

*We went through the pregnancy stage, so everyone has kids at home. I find hardly the time doing some physical activities. So maybe something that gives like, let’s say every second day half an hour that I could do even if my kids are around if it’s in your house…*

Potential benefits and drawbacks to pedometer (step count monitor) use were addressed (Theme 7: Perceived Advantages/Disadvantages on the use of Pedometers and Support for Physical Activity as Subthemes). Most viewed the pedometer as a potentially-motivating tool to maintain or increase physical activity levels:

*…walking is the easiest exercise you can do – because you do it, to go to the bathroom, to clean the house. …and now if you have something that tracks it that tells you, ‘ok, it’s not too bad*

Some, however, were concerned that they would be discouraged if step counts were low, and suggested that a pedometer would not address their lack of motivation:

*For me, it does not change anything because I am always in a car. I walk very little so I will feel even guilty for not having walked. I will look down at the low numbers and I’ll feel anxious*

As for food intake, support from other individuals was needed to achieve and maintain higher activity levels:

*I like having a buddy system. I’ve never liked to do exercise on my own… I need someone there to help me to motivate me, I can’t go there alone.*

*I’m telling you, if I walk with the group here, if there’s another large person like me, then I’m going to go.*

QUESTION 2: What would prevent someone like you from taking part in a program like this?

The two major barriers that emerged were lack of family support (Theme 8) and of time (Theme 9). Some participants reiterated (see Theme 3) that partners often did not know how to be supportive with DM2 prevention efforts, and that the ideal program would help their partners achieve this understanding.

*I just thought that our husbands or mates- not that they don’t want us to be healthy and learn about this - but they also are feeling time constraints. Maybe if they had an information session at the beginning to underline how important this is… what it’s going to entail, that they might have to give up a little bit of their time for us to do that. They can ask questions, they can learn a little, and also that they know what their spouses or their mates are doing in that time that they’re away*

Time constraints were an even more important barrier to attend a DM2 prevention program, because of competing responsibilities related to children and employment.

*Time constraint is a big one. Like with people with kids, I know I can’t with a drop of a dime just take off and go somewhere*

QUESTION 3: What would facilitate your planning around meal preparation?

Participants expressed interest in developing the knowledge and skills to increase the variety of their dietary intake, with an emphasis on budgetary considerations, time efficiency, and educational tools (Theme 10):

*-Try to use the same broccoli several times…I would like to be able to make different recipes with it in the days that follow.*

*-…sometimes I see recipes which seem great, there are vegetables, fruits, which cost the skin off your back and are NOT on special. I won’t make those recipes because they don’t fit into my budget.*

*-Quick meals. Five o’clock and I want to eat by six o’clock, let’s start!*

Planning ahead was viewed as a potential means of saving time, and participants suggested that any program should emphasize planning strategies. Several made reference to planning in order to reduce the number of trips per week to grocery stores. Diabetes-friendly recipe books were thought to be useful tools. Others felt that familiarizing themselves with grocery store products would enable them to make better choices. Participants stressed the importance of cultural preferences when planning meals (Theme 11), with a need for information on nutritional content of foods specific to their culture.

*…you know it’s not part of the food guide but the reality is there are people who are Indian, Italian, Greek and you can’t change your whole family at once…What are the things that I can change without changing the culture of the food? What are the things that you can limit so your family doesn’t feel like they can no longer eat what they like?*

One of the probes that we integrated queried attitudes about using meal replacement products as a means of controlling dietary intake, particularly as meal preparation skills were improved. The idea would be to return to consumption of regular meals, tapering meal replacement consumption,

The discussion that followed this probe suggested ambivalence and/or lack of endorsement for meal replacement use (Theme 12). A few participants viewed breakfast as a challenge, and an opportunity for consuming shakes.

*I’m hungry, I take a shake, it’s a substitute I take on four out of five mornings before doing my morning shifts and I can say, “I ate well, I have all the nutrients I need to make it till lunch*

Others considered meal replacement as a healthier way to cope with cravings, compared to less healthy snacks. However, more barriers than advantages to meal replacement use were highlighted. Many participants were concerned that meal replacement use would constitute a poor example of eating behavior to their children. Others feared that meal replacement use could foster poor dietary habits.

*It’s not setting an example if we say, “I’m going to have a shake and you go and organize your own dinners.*

*I’m against it because I have a teenager. Every morning, I must argue with her. At this moment, in her adolescence, she has an aversion to food. She refuses to eat because she doesn’t want to gain weight… all she wants to eat are those bars whereas I am trying to get her to eat a real breakfast before she goes to school…*

QUESTION 4: What would facilitate your interactions?

This question was prefaced by the moderator with the following statement: “One of the benefits of programs like the ones we are studying is that people have a chance to share ideas with each other. Some people form friendships and even get together outside the sessions. This helps to support one another in making healthy lifestyle changes. Now, I would like you to think about interacting with other women in your position.” The moderator specifically probed the role of social media options.

The discussions that addressed social interactions (Theme 13) revealed the perspective that ‘getting out’ and having face-to-face contact with others were important to an overall sense of well-being. Social media, such as web-based groups, was perceived to be a useful tool for planning events, receiving and offering encouragement, and sharing knowledge.

*For example, you develop something on the web and send an e-mail to the women who participated… And we can respond to others and say, for example, “I live in (location), you live here. Maybe we can find a weekend where we go walking together or we cook together because I think we need someone’s support*

For others, the use of social media was not appealing, even as a complementary tool to the program and having to use the computer to interact was described as being tedious.

*-You know, I work in front of the computer all day. Naturally, I don’t feel like sitting in front of the computer in the evenings…For me the contact, the sharing is more interesting in person than in front of the computer.*

*-It would help but I think seeing the person because people can write whatever they want on the web – I lost 10 lbs…, you know it’s seeing that person, seeing the effect of the exercise helps.*

Aside from social media, other proposals to facilitate social interaction were suggested, such as the idea of a ‘buddy system’, and including children on social occasions.

*-It would be nice if we could partner up people who live close to each other and sort of make a goal. We actually live pretty close together and it would be nice to have between the two of us a cooking night together.*

*-If they have kids of the same age, for example. That way, we can see each other; do something with the kids because normally, we spend most of our free time with the kids…*

### Conclusion

Among women with prior GDM who attended a focus group discussion, there was a clearly-expressed need for social support- from family, from health professionals, and from peers - to achieve changes in eating and physical activity behaviours. Moreover, while time constraints were a critical barrier to overcome, face-to-face interactions with peers and professionals were deemed essential. Social media could provide an important- but adjunctive- role. Most importantly, family-related considerations appear to be critical in designing DM2 prevention programs. Explicit involvement of partners in a program would not only increase the social support for behavioural change, but also specifically help to achieve buy-in for changes in the home food environment. Attendance at face-to-face sessions required integration of childcare, as child-related responsibilities would constitute an important barrier to attendance, particularly if both partners were to attend. Our participants recognized that a DM2 prevention program could lead not only to health benefits for themselves, but also for their children, and this was underscored as a motivating factor. Moreover, meal replacements were viewed as undesirable tool, largely because of perceptions that their use could distort children’s concepts of healthy eating.

The importance of social support for behavioural change highlighted by our participants is consistent with prior studies. Social support from family members and friends has been demonstrated to enhance physical activity levels among women in general [19], [20]. While our participants expressed a need for face-to-face interactions, they did acknowledge that social media could have a role in this regard. In a previous qualitative study of women with a GDM history [14] greater support for an Internet-based approach was suggested than identified in our study; however, in this previous study, recruitment was partly through the Internet, perhaps accounting for a selection of individuals with a preference for this form of intervention delivery.

Our participants’ assertion that altering family eating habits requires their partners’ involvement and support is consistent with a previous study indicating that household taste preferences may impede adoption of healthier dietary choices [21]. In fact, dietary interventions delivered to one spouse have been demonstrated to have effects on the partner (i.e., weight loss), even when only one partner is involved in the intervention [22]; [23]. Further, the offspring of women with a GDM history are at higher risk for insulin resistance and obesity [24]–[26], and thus may benefit importantly from improvements in family eating habits.

Notwithstanding the substantial increased risk for DM2 following a GDM pregnancy, few women within five years of a GDM diagnosis appear to be willing or able to engage in a group discussion of potential strategies, as suggested by the 10% response rate to our invitation. This somewhat limits the generalizability of our findings to the wider population of women with prior GDM, although it likely captures the needs and preferences of those seeking assistance with DM2 prevention. Among those who explicitly indicated they could not participate, time constraints and child-related responsibilities were factors. Language-related barriers may have been important for some women. Although we did have representation from women from several regions of the world, there was a notable absence of women of South Asian ancestry, despite the fact that women from this ethno-cultural group constitute an important proportion of women seen and diagnosed with GDM at antenatal clinics in Montreal [27]. Lack of acknowledgement of personal DM2 risk may have also affected participation rates; a previous study indicates that although 90% of women with a GDM history are aware that GDM is an indicator for future development of DM2, fewer than 20% view themselves to be at risk [28]. Finally, some women may acknowledge risk but lack self-efficacy to achieve changes in eating habits and physical activity levels [29], [30]. There is likely a need to better impress upon women with a GDM history that they themselves are at risk for DM2, that the risk data are not simply impersonal statistics, and that there are proven strategies to help them reduce this risk, with potential health benefits for themselves and their families.

Our participants voiced a need for active involvement of their partners in a DM2 prevention program. An element of face-to-face interactions with professionals and peers is needed to maximize knowledge and support, but childcare is critical to allow session attendance. We are presently launching a pilot study that will include four (once/month) in-person hands-on meal preparation/educational sessions, with on-site childcare, and partner attendance at two of the four sessions. Support between-sessions will include telephone calls from study personnel and a dedicated website with tips, tools, and opportunities for interactions among participants. We will examine effects on anthropometric measures and insulin sensitivity. A GDM diagnosis provides a window of opportunity for DM2 prevention. If the mother and other family members are appropriately engaged, the net effect may be reduced risk not only for the mother but also for her partner and children.

## Supporting Information

Appendix S1 Illustrative quotations by question and theme.
(DOCX)
