# Supplementary material for: Strategies to Optimize Participation in Diabetes Prevention Programs following Gestational Diabetes: A Focus Group Study
Source: PLoS One. 2013 Jul 4;8(7):e67878. doi: 10.1371/journal.pone.0067878 (PMC3701629; doi:10.1371/journal.pone.0067878)
Supplement: Appendix S1 — Illustrative quotations by question and theme. (DOCX) [file pone.0067878.s001.docx]

**Appendix 1. Illustrative quotations by question and theme**

| **QUESTION 1: What would make someone like you take part in a program like this?** |
| --- |
|  |
| **Theme 1: Potential Benefits from Nutrition/Culinary Education** |
| *In the nutritional packaging part, it says fiber, insoluble, soluble. What’s the difference?* |
|  |
| *The nutritionist would be able to tell us what we are eating and whether it will help us* |
|  |
| *Do you follow strictly (the plan) like you were pregnant or do you deviate from it a little because your body’s handling it differently… Am I going in the right direction?* |
|  |
|  |
| **Theme 2: Support for Life Style Change and Maintenance** |
| ***Subtheme: Prevention of diabetes*** |
| *They told me that once you deliver your baby, the chances of diabetes coming back is low. However, I have since then become aware that it is not true. My sister was diagnosed in her 50s, another woman I know as well* |
|  |
| *It is dormant, and that’s it. The older you get, the closer you get to forty – it comes out. You don’t realize it. But I realize that, my lifestyle has to change* |
|  |
| *For me, it is to try to prevent that I experience diabetes. It is in my family* |
|  |
| *For me, it is to prevent diabetes given that I have a predisposition. My father has diabetes. It’s hereditary* |
|  |
| ***Subtheme: Availability of support system.*** |
| *From different women to get ideas also. How they maintain their diet, and so forth* |
|  |
| *You need to get out, you need to go do stuff for yourself. I think part of the motivation that will get you kick-started is if you have people to talk to* |
|  |
| *When you go to the gym, you have your personal trainer to tell us what to do and you follow it. We need a personal trainer. Help us keep going after we give birth, not to fall back. After so many months of doing it then maybe we will stay motivated* |
|  |
| *During the pregnancy, we have the diabetes and so we were very closely watched. Then after, it’s very easy to fall back into the habit that we had before. So let’s say there is a group that we go we learn the good things. It’s very hard to keep in the track...* |
|  |
|  |
| **Theme 3: Family Participation** |
| *In fact, if the husbands were involved that would make my participation 100%!* |
|  |
| *At our house, I’m the chicken breast salad. And my husband is still buying hotdogs. If you send him to the grocery store – it’s a disaster!* |
|  |
| *It’s nice that we’re being educated. I can tell the message to my husband. He can be supportive- but when I give this man brown rice, he looks at me funny. So I can explain to him really what’s going on but if he would hear it from elsewhere, maybe, it’ll be different.* |
|  |
| *I think the kids too it’s important to learn from a young age, the proper eating habits. You might have something and it doesn’t always have to be sweet. Otherwise, later on, she’ll have diabetes. So I think it’s very important* |
|  |
| *No medical doctor is going to motivate me, but my daughter. She prevents me from eating what I should not be eating* |
|  |
|  |
| **Theme 4: Right Timing/Opportunity for Intervention, with Intervention Dependent on Life Cycle and Individualized Schedule as Subthemes** |
| ***Subtheme 1: Intervention dependent on life cycle.*** |
| *When we are pregnant we have to come in every week and if we are also part of such a group before we give birth, we will already have this sense of belonging which could then continue following the birth* |
|  |
| *When women have their prenatal classes could be an option as this would ensure that their husband would also hear the same thing* |
|  |
| *For me, it is better if it’s earlier because after I give birth I had my blood test and everything was fine. I lost my pregnancy weight and when I returned to eat, I gained more weight than I had before I was pregnant. It wasn’t good. I had to have someone actually tell me, you know you still need to eat x amount of food and all that* |
|  |
| *Once you have your baby, you’re followed up and then you’re there to fend for yourself. Do I still eat the way I’m told? There are no resources given to you after* |
|  |
| ***Subtheme 2: Individualized schedule.*** |
| *You’re in the routine of being ‘mommy’ and feeding him and taking care of him. In my case, there really wasn’t much time or anything else of support, and I think even if there was support, I would have found it hard to do it. Like he wasn’t the type of baby that would eat every 2 hours, it was every 4 hours so there was still some time but then you’re so tired. And you still have stuff to do* |
|  |
| *I think the flexibility of a schedule. On a weekday, on a weekend because you don’t really know people’s individual schedules, there’s a possibility of making it flexible, then maybe people can pick and choose a daytime, an evening, a weekend. Whatever suits them* |
|  |
|  |
| **Theme 5: Availability of Child Care** |
| *You could offer services where you bring a child with you in a daycare or workshop, where you know that they are being taken care of*. |
|  |
| *Have a place where we can bring our children for someone to watch them so that we can attend the program without having to worry about them* |
|  |
| *If we know we have a baby sitter. It’s a lot easier*! |
|  |
|  |
| **Theme 6: Awareness of Physical Activity Integration** |
| *How you can incorporate physical activity on a day-to-day basis if you don’t have a lot of time? How you can do it at home to facilitate…* |
|  |
| *We went through the pregnancy stage, so everyone has kids at home. I find hardly the time doing some physical activities. So maybe something that gives like, let’s say every second day half an hour that I could do even if my kids are around if it’s in your house*… |
|  |
| *I don’t know if the program could offer an aerobic or it could be something like being able to do it outside or inside* |
|  |
| *We would like to have a series of exercises that we can do at home if we cannot go out* |
|  |
| *When you mentioned a trainer that would have been good for me but I wouldn’t be able to afford one 3 times a week. Once you get into the habit, they can teach you exercises of how to do it at home with what you have as well. Not necessarily in the gym with the dumbbells and stuff like that* |
|  |
|  |
| **Theme 7: Perceived Advantages/Disadvantages on the use of Pedometers** |
|  |
| *...walking is the easiest exercise you can do – because you do it, to go to the bathroom, to clean the house. ...and now if you have something that tracks it that tells you, ‘ok, it’s not too bad* |
|  |
| *To monitor to say, “Ok, did I reach my target today? Maximize it more the next day....balances it off for the whole week* |
|  |
| *Yes,* (on using a pedometer) *because there was a period where I was sedentary...I would like to control this better* |
|  |
| *It’s a good tool but I know I feel it’s a lot of pressure on the individual* |
|  |
| *For me, it does not change anything because I am always in a car. I walk very little so I will feel even guilty for not having walked. I will look down at the low numbers and I’ll feel anxious* |
|  |
| *I don’t have the time, they* (referring to her children) *don’t fall asleep till 10:30 or 11 and then I think I would just be disappointed* |
|  |
| ***Subtheme: Support for Physical Activity*** |
| *I like having a buddy system. I’ve never liked to do exercise on my own... I need someone there to help me to motivate me, I can’t go there alone* |
|  |
| *I’m telling you, if I walk with the group here, if there’s another large person like me, then I’m going to go* |
|  |
| *The thing is that to help move forward from not being motivated is to say to motivate yourself maybe when we have this program, when this program exists, is to say, it’ll be good based on evaluation of your body size, whatever, that you should maintain a certain number of steps for instance. A target. So that way it motivates you* |
|  |
| *A follow up or you know, at least to encourage you. Because you always feel if you don’t get this done, it’s discouraging* |
|  |
| **QUESTION 2: What would prevent someone like you from taking part in a program like this?** |
|  |
| **Theme 8: Lack of Family Support** |
| *I just thought that our husbands or mates- not that they don’t want us to be healthy and learn about this - but they also are feeling time constraints. Maybe if they had an information session at the beginning to underline how important this is… what it’s going to entail, that they might have to give up a little bit of their time for us to do that. They can ask questions, they can learn a little, and also that they know what their spouses or their mates are doing in that time that they’re away* |
|  |
| *They might have to give up some of their spare time in order for us to come and sometimes they are feeling too just like we feel that our lives are changing, and you suddenly don’t have time to go and play hockey, and do this or do that. So maybe help them be a little bit more understanding* |
|  |
| *It’s a disaster– so if the husbands were involved, I would love that* |
|  |
| *My husband doesn’t help too well* |
|  |
| *No, I’d cook for myself and my daughter. So he could go fend for himself. He wouldn’t like it anyways. So there’s no point* |
|  |
|  |
| **Theme 9: Lack of Time** |
| *Well, you can never depend on things that kids have going on. They’ve got hockey..* |
|  |
| *Time constraint is a big one. Like with people with kids, I know I can’t with a drop of a dime just take off and go somewhere* |
|  |
| *On Saturdays there are the children’s activities...* |
|  |
| *If you have a job that doesn’t allow you to be flexible* |
|  |
| *Homework. If we had a journal or something to track everything that we were eating, that sort of thing. Then it would sort of be “Oh, I forgot”, you know what I mean? If we had to track a lot, you know* |
|  |
| **QUESTION 3. What would facilitate your planning around meal preparation?** |
| **Theme 10: Planning Meals with Diversity, Budget, Time-efficiency, and Educational Tools**  **as Subthemes** |
|  |
| ***Subtheme 1: Diversity*** |
| *It would be nice to have, you know, replacements. You know, if you don’t want to cook rice you could do quinoa or barley* |
|  |
| *Try to use the same broccoli several times...I would like to be able to make different recipes with it in the days that follow* |
|  |
| ***Subtheme 2: Budget*** |
| *...sometimes I see recipes which seem great, there are vegetables, fruits, which cost the skin off your back and are NOT on special. I won’t make those recipes because they don’t fit into my budget* |
|  |
| *I do try to look at the flyers on Wednesdays and I uh get what’s on sale… then I try to figure out what I’m going to make around that* |
|  |
| *Well, that (referring flyers on grocery specials)* *might help also with the budget because you know that’s the special of the week… and you can do a variety of menus* |
|  |
| ***Subtheme 3: Time-efficiency*** |
| *Quick meals. Five o’clock and I want to eat by six o’clock, let’s start!* |
|  |
| *Easy meals...20 minutes would be reasonable* |
|  |
| *I would like to see cooking in bulk and freezability* |
|  |
| *Cook once per week, one time per week for the WHOLE week* |
|  |
| *During the week, I want to know what I’m going to do. I don’t want to go twice in a week to do grocery* |
|  |
|  |
| **Theme 11: Integrating Cultural Considerations** |
| *…the challenge is culture is often forgotten. I see the food but then it’s like my family, we know that we’ve grown this way and this is how they like to eat...how do you transition your whole family to change?* *...you know it’s not part of the food guide but the reality is there are people who are Indian, Italian, Greek and you can’t change your whole family at once...What are the things that I can change without changing the culture of the food? What are the things that you can limit so your family doesn’t feel like they can no longer eat what they like?* |
|  |
| *We come from Mexico and we eat tortillas...will there be people to help teach me about those ingredients, those products?...Sometimes we don’t know what the nutritional content is!* |
|  |
|  |
| **Theme 12: Views on Meal Replacements with Perceived Advantages of using Meal Replacements and Barriers to using Meal Replacements as Subthemes** |
|  |
| ***Subtheme 1: Perceived advantage of meal replacements*** |
| *... one of my big problems is breakfast and I actually love the shakes because I have a very small amount of time to get all the kids ready and to get myself ready and get out for work.* |
|  |
| *I’m hungry, I take a shake, it’s a substitute I take on four out of five mornings before doing my morning shifts and I can say, “I ate well, I have all the nutrients I need to make it till lunch* |
|  |
| *I’d rather have something nutritious which replaces a meal rather than going to get chocolate bars, pastries, cakes to calm my hunger* |
|  |
| ***Subtheme 2: Perceived barriers to meal replacements*** |
| *It can be done individually but when we have a family, I don’t think we can do this with the whole family* |
|  |
| *It’s not setting an example if we say, “I’m going to have a shake and you go and organize your own dinners* |
|  |
| *I’m against it because I have a teenager. Every morning, I must argue with her. At this moment, in her adolescence, she has an aversion to food. She refuses to eat because she doesn’t want to gain weight... all she wants to eat are those bars whereas I am trying to get her to eat a real breakfast before she goes to school…* |
|  |
| *I’d rather jump into it* (talking about cooking) *because then you will get lazy and I’ll always keep one of those meals”* AND “*I buy the box, I am embarrassed to tell you, but I will eat all of them in one day. I have no control...* |
|  |
| *Show* us *how to cook my meals, show me what I could do different* |
|  |
| *I don’t see how that would benefit. I would use that meal replacement as my lunch. At lunch, I don’t have an issue, I enjoy my sandwiches* |
|  |
| **QUESTION 4: What would facilitate your interactions?** |
| *Statement prior to Question 4:* One of the benefits of programs like the ones we are studying is that people have a chance to share ideas with each other. Some people form friendships and even get together outside the sessions. This helps to support one another in making healthy lifestyle changes. Now, I would like you to think about interacting with other women in your position.  *Probe:* What about social media options? |
|  |
| **Theme 13: Facilitators of Social Interactions** |
|  |
| ***Subtheme 1: Social media as facilitator to social interactions*** |
| *For example, you develop something on the web and send an e-mail to the women who participated... And we can respond to others and say, for example, “I live in* (location)*, you live here. Maybe we can find a weekend where we go walking together or we cook together because I think we need someone’s support* |
|  |
| *I think it would help knowing that we don’t feel alone... Other mothers are working. Let’s face it we feel guilty working, you’re not home and you don’t prepare well the house meal. I think it would help knowing of others like that* |
|  |
| *If you’re supporting each other and you post, you know I just did 30 minutes without any music like, “I’m a hero”, you know?? (giggles), and then you know, it’s encouraging* |
|  |
| *Our point of reference....We are part of a group and can search for recipes, information, contacts...* |
|  |
| ***Subtheme 2: Negative views of social media***. |
| *You know, I work in front of the computer all day. Naturally, I don’t feel like sitting in front of the computer in the evenings...For me the contact, the sharing is more interesting in person than in front of the computer* |
|  |
| *I’m part of a couple… no need to exchange recipes. I just don’t think about it. Because by the time you sit down and actually look at the computer, it’s like 8 or 9 at night, you’re like oh I’d rather see what this person’s doing and then you’re tired and you go to bed!* |
|  |
| *the reason I will go somewhere is because I like to be in the company of people. On the internet, I’ll listen, I’ll exchange but I won’t “act” very much* |
|  |
| *It would help but I think seeing the person because people can write whatever they want on the web – I lost 10 lbs…, you know it’s seeing that person, seeing the effect of the exercise helps.* |
|  |
| ***Subtheme 3: Alternative forms of social interaction*** |
| *It would be nice if we could partner up people who live close to each other and sort of make a goal. We actually live pretty close together and it would be nice to have between the two of us a cooking night together.* |
|  |
| *If they have kids of the same age, for example. That way, we can see each other; do something with the kids because normally, we spend most of our free time with the kids...* |
|  |
| *Why can’t it be like a number where we can call somebody that’s available at this time to this time to talk about, ‘OK I’ve got the diabetes, I need to know what if I do this or could I do this. Can you give me any suggestions?* |
|  |
| …*either a help line with a nutritionist or a dietitian* |
|  |
